# Do HIV provider and client perspectives align on person-centered care? Lessons learned from implementation of the Person-Centered Care Assessment Tool (PCC-AT) in HIV treatment settings in Ghana

**DOI:** 10.1371/journal.pgph.0003457

**Published:** 2024-09-06

**Authors:** Jessica E. Posner, Malia Duffy, Caitlin Madevu-Matson, Henry Tagoe, Amy Casella, Melissa Sharer, Henry Nagai

**Affiliations:** 1 John Snow, Inc., Washington, DC, United States of America; 2 Health Across Humanity, LLC., Boston, Massachusetts, United States of America; 3 John Snow, Inc., Boston, Massachusetts, United States of America; 4 John Snow, Inc., Accra, Ghana; 5 Saint Ambrose University, Davenport, Illinois, United States of America; University of Washington Seattle Campus: University of Washington, UNITED STATES OF AMERICA

## Abstract

Person-centered care (PCC) is foundational to improve client’s experiences in care while advancing HIV-related outcomes. However, information is scarce on how to assess PCC in HIV treatment settings. This study team developed the PCC assessment tool (PCC-AT) to assess the performance in HIV clinics in Ghana. The objectives of this study were to: (1) pilot the PCC-AT and assess scoring consistency and reliability among clients and providers; and (2) assess content validity of the PCC-AT through client key informant perspectives and experiences. An analysis of similarities and differences in PCC-AT domain scores between ART providers and clients was conducted to assess score reliability. Axial and open coding of transcripts using NVivo identified key themes. Findings indicate that the PCC framework aligns with client’s priorities, additionally two out of the three PCC domain scores demonstrated consistency between ART providers and clients. Emerging differences in ART provider and client perspectives highlighted opportunities for growth and underscored the importance of continually gathering client feedback as an integral component of a PCC assessment to continually strengthen ART services.

## Introduction

In Ghana, 87% of persons diagnosed with HIV are on antiretroviral treatment (ART). Ghana’s ‘treat all’ policy, elaborated within the National HIV and AIDS Strategic Plan 2021–2025, has not yet been fully optimized, due in part, to unique access and retention barriers that various populations experience [1–3]. Among key populations, criminalization, stigma, fear of disclosure of HIV and sexual minority status, and poor treatment from healthcare workers contribute to low health seeking behaviors [4–6]. For men, harmful masculinity norms contribute to reduced HIV treatment access while women experience challenges associated with socioeconomic, religious and cultural factors, poor treatment from healthcare workers, and costs [7, 8]. Among adolescents, fear of disclosure and stigma as well as financial challenges present HIV treatment access barriers [9] while younger children experience adherence and viral load (VL) suppression challenges due to inconsistent caregivers and difficult to administer ART formulations [10].

Person-centered HIV treatment services that are tailored to the needs and expectations of diverse populations offer an important opportunity to improve client experiences in care as well as HIV treatment access, uptake and continuity while advancing progress towards universal treatment and epidemic control [11–13]. The International AIDS Society defines person-centered care (PCC) as “…a multidisciplinary, integrated and long-term focused approach to care for people living with and affected by HIV that is responsive to their evolving needs, priorities and preferences [14].” The WHO defines integrated people-centered services as "putting people and communities, not diseases, at the center of health systems, and empowering people to take charge of their own health rather than being passive recipients of care.” [15]

Models of PCC in HIV treatment settings that have emerged in the literature focus on ensuring services are inclusive of diverse populations while increasing the convenience through differentiated treatment delivery models such as multi-month dispensing of ART, community-based ART pick-up and home delivery, extended and special service hours for diverse populations, tele-medicine, individual and group adherence support, and task shifting. These models have demonstrated increased treatment initiation, adherence, retention and viral suppression and contributed to reduced gender disparity gaps [3, 13, 16–19].

In Ghana, emerging data from interviews with people living with HIV (PLHIV) have identified some priorities for PCC including attending to the individual physical, psychological, social, and spiritual welfare of clients; support with safe fertility and sero-discordant relationships; and greater participation in healthcare decision making [4]. However, language and unequal power dynamics may inhibit clients from sharing their concerns with health providers [11]. While patient reported experience measures provide important measures to assess client’s perspectives [20], they offer a limited perspective on the organizational and technical considerations that are required to implement PCC and there is scarce information available on how to assess PCC services in HIV treatment settings.

**Fig 1:**
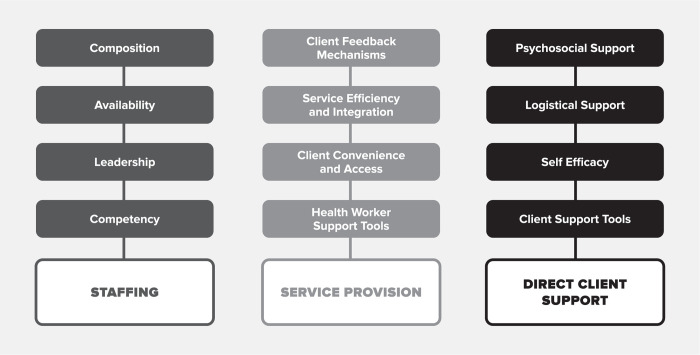
Framework for PCC in HIV treatment settings.

This study team sought to fill this gap by applying the person-centered care assessment tool (PCC-AT) to assess the degree to which clinics in Ghana deliver PCC. The PCC-AT was informed by a PCC framework (Fig 1), published elsewhere [21]. The Excel-based PCC-AT measures ART providers’ perspectives on PCC service delivery within HIV treatment settings using 56 discrete performance expectations across 12 subdomains. While the PCC-AT has been reviewed by public health experts and clinicians, this is the first time it has been piloted in HIV treatment settings with the specific aim to understand how clients perceive and value the tool.

A full description of each domain and sub-domain is available in S1 File.

The objectives of this study were to:

1. Pilot the PCC-AT and assess scoring consistency and reliability through determining if the PCC-AT scores, identified by ART providers, match the perspectives and experiences of PLHIV key informants who access services at each study health facility.
2. Assess content validity of the subdomains and domains within the PCC-AT through gathering information from clients regarding their perspectives and experiences accessing HIV treatment services.

These findings complement a PCC-AT feasibility study published elsewhere [22].

## Materials and methods

This study employed a mixed method, cross-sectional study design consisting of a pilot PCC-AT, focus group discussions (FGDs) and key informant interviews (KIIs).

### Study setting

The USAID Strengthening the Continuum of Care project in Ghana partners works in partnership with the government of Ghana to implement strategies including index testing, contact tracing and testing, targeted outreach testing, and use of case managers to identify, link and support clients on ART. The project was selected for this study due to: 1) availability of clinical sites providing direct HIV treatment services; 2) staff interest to scale-up PCC to strengthen services; and 3) availability of staff already knowledgeable about the PCC-AT given their participation in development of the PCC framework and PCC-AT. Five health facilities in western Ghana were purposively sampled to represent geographical and size diversity in the levels of health facilities providing HIV treatment service in Ghana. Facilities included a Regional Hospital, two Government Hospitals, one Health Center and a Quasi-Government (military) health facility which also serves civilians. This mix of facilities covers urban, parastatal, and community settings. Please see S2 File for further information on study facility characteristics.

### Study population and inclusion criteria

#### Health facility staff

The study team aimed to have a minimum of seven health facility staff members participate in each PCC-AT pilot. The sample-unit for each facility is the facility’s ART team members and participant selection was purposive based upon their knowledge and availability to participate. Facility ART providers included nurses, lab technicians, counselors, pharmacists/dispensing technicians, data managers/officers, and community group liaisons whose work at the facility includes provision of HIV services. Inclusion criteria prioritized ART providers with at least one year of experience providing HIV services to clients in the facility.

#### Clients

Recruitment of PLHIV for key informant interviews (KIIs) was done using a convenience approach from a list of clients attending ART/HIV care services on the day of data collection. Clients were invited to participate if they met the following criteria: greater than 18 years old, diagnosed with HIV, received ART/HIV services on the day of data collection, confirmed that they had attended at least one appointment at the facility prior to the day of the visit, and willing to provide informed consent. On the day of data collection, we noted commonalities between client responses to estimate how large a sample of clients were needed per facility to reach saturation.

### Design and measure construct

#### Scoring consistency and reliability

The study team asked clients during KIIs to assign a performance score to each domain (1–4; 1 = very poor, 4 = very good) after sharing their perspectives on service delivery for each subdomain. We conducted an analysis of similarities and differences in PCC-AT domain scores between ART providers and clients to assess reliability of PCC-AT scores and to determine if scores assigned by ART providers would be similar to those assigned by clients.

#### Content validity

The study team gathered insights from clients via KII using open-ended questions to ensure the content included in the PCC framework (which the PCC-AT is based upon) accurately encapsulates PCC delivery, capturing the experiences and knowledge from expert clients with lived experience. Using a KII guide (S3 File), the study team gathered insights on key informants’ perspectives of their service experiences in-line with each PCC framework subdomain. Key informants were then asked to express their degree of agreement regarding the importance of each PCC subdomain as it relates to HIV treatment service delivery (1–4; 1 = strongly disagree, 4 = strongly agree). Clients were also asked to describe actions the facility could take within each subdomain to improve their experiences of PCC.

### PCC-AT procedures

PCC-AT tool implementation at health facilities was conducted by two full-time English and Twi/Fante speaking data collectors. All data collectors underwent a two-day training led by the PCC-AT developers, consisting of an introduction to the purpose and nature of the study, instruction on obtaining informed consent, interviewing and facilitation techniques, data storage, and practice implementing the PCC-AT. A lead facilitator was designated responsible for introducing the study, collecting informed consent, and facilitating the PCC-AT process with the health facility team; while a second data collector took responsibility for managing PCC-AT inputs, scoring, and observational note-taking.

Facilities were contacted prior to the data collection period to confirm likely health facility staff and client availability; however, official recruitment took place on the data collection day at each facility between May 15^th^ and 18^th^, 2023. Prior to commencing each PCC-AT, the study team distributed a study summary sheet and obtained written consent from each health facility participant which was signed in the presence of a self-selected witness. Following consent, the lead facilitator guided PCC-AT implementation to enable ART providers to assess their own strengths and weaknesses through discussing each performance expectation, coded using a specified benchmarking approach (e.g., yes/no or all/mostly/partly/none). Each performance expectation was linked to an ordinal score, from which geometric means were calculated and rounded down to the nearest whole number to elicit subdomain scores (1–4; 1 = standard never met to 4 = standard always met). Subdomain scores were then averaged to determine domain scores (1–4; 1 = inadequate capacity to 4 = strong capacity). The assessment process was intended to promote information sharing, a healthy internal dialogue, and consensus building by including a range of ART provider team members who could discuss institutional abilities, systems, procedures, and policies that influence PCC across subdomain performance expectations.

### Informed consent

Prior to each KII, the study team read the study summary sheet and informed consent sheet in the language preferred by the client (English, Twi, Fante). Each informed consent sheet was signed in the presence of a self-selected witness.

### KII procedures

Using the KII guide, a study team member gathered information on client experiences and perspectives within the PCC framework domains and subdomains, then asked the client to assign a performance score based upon their experiences at the health facility for each domain. KIIs were conducted either in the Akan language (Twi/Fante) or in English based upon the client’s preference. Interviews were recorded, transcribed, and then translated into English.

### Data analysis

#### Quantitative

Quantitative analysis included information collected on how many and which ART provider team members (by cadre) participated in the PCC-AT pilot, their years of experience at the health facility, and gender. For KII participants, information was collected on total time accessing treatment services at the study health facility, years since diagnosis, age and gender. Further quantitative analysis included comparisons between ART provider-rated facility scores and aggregate client scores for each PCC-AT domain. Spearman’s rank correlation (r_s_) was used to assess the relationship between ART providers and client scores, which were calculated as mean values for each facility.

#### Qualitative analysis

KII transcription data was analyzed using descriptive coding with NVivo software. Axial and open coding of the transcript text allowed for deconstruction of the text and uncovered emergent themes, concepts, issues, and ideas. Initial transcripts were coded separately by two members of the study team according to the framework and compared to ensure intercoder reliability.

### Data security

Participant confidentiality was protected by, 1) not including any names of participants (ART providers or clients) on any data collection materials, 2) storing data in a secure place, 3) developing codes during qualitative data analysis so that any potential identifiers were not associated with data, and 4) maintaining strict adherence to the principles of voluntary participation, confidentiality, anonymity and protection of human subjects as guaranteed by the consent form.

### Ethical approval and protocol registration

The study received ethical approval from JSI’s IRB (IRB #22-53E) and Ghana Health Service Ethics Review Committee (Protocol ID NO: GHS-ERC 008/10/22). The protocol was registered with OSF prior to data collection (https://doi.org/10.17605/OSF.IO/763M4).

## Results

### Study participant characteristics

#### Study participants

Across the five study facilities there were a total of 37 participants with seventy percent of study participants female (Table 1). The mean years of experience was 4.7 (±3.9) years with most participants (73%) having between 1–5 years of experience. Sixty percent of the study participants were Nurses, followed by Laboratory Scientist/Technicians and Data Officers (14%) and Pharmacist/Pharmacist Technicians (11%).

**Table 1:** Participant characteristics.

Characteristic	Frequency (%)
**Gender**	
Female	26 (70.3)
Male	11 (29.7)
**Years of experience at facility**	
**Mean (SD)**	**4.7 (±3.9)**
<1 year	2 (5.4)
1–5 years	25 (67.6)
6–10 years	7 (18.9)
11–15 years	3 (8.1)
**Total**	**37 (100.0)**

#### Client key informants

Four clients participated from each facility for a total of twenty KIIs. A total of 85% of key informants were female (n = 17) and 15% were male (n = 3) with ages ranging from 24 to 59 years, with a mean of 41 years (Table 2). Treatment start year at the facility ranged between 2006 and 2023, with a mean start year of 2017.

**Table 2:** Client characteristics.

Characteristic	Frequency (%)
**Gender**	
Female	17 (85)
Male	3 (15)
**Age** *	
**Mean (SD)**	**41.4 (±10.8)**
24–33 years	5 (25)
34–43 years	8 (40)
44–53 years	1 (5)
54+ years	5 (25)
**Treatment start year at facility**	
**Mean (SD)**	**2017 (±4.4)**
2006–2010	2 (10)
2011–2015	5 (25)
2016–2020	9 (45)
2021+	4 (20)
**Total**	**20 (100.0)**

* One client did not disclose their age

### PCC-AT scoring consistency and reliability

#### PCC-AT score comparison

**Fig 2:**
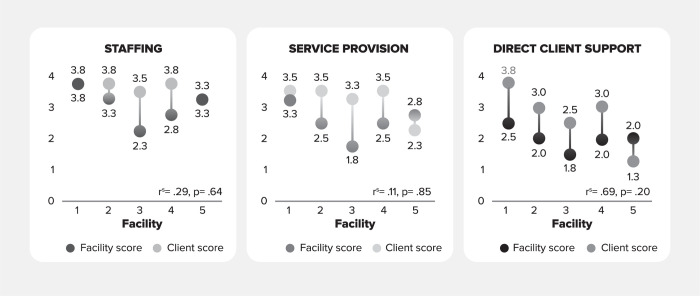
Comparison of PCC-AT domain scores, clients vs. ART providers.

Using overall averages across facilities, ART providers rated their facilities’ performance highest in the staffing domain (3.1), followed by service provision (2.6), and direct client support (2.0). Similarly, clients rated their facilities’ performance highest in the staffing domain (3.6), followed by service provision (3.2), and direct client support (2.7). As shown in Fig 2, across facilities one to five, scores assigned by ART providers were most often equal to or lower than average scores assigned by clients, but with a small sample size, the differences were not statistically significant.

### Content validity of the PCC framework

**Fig 3:**
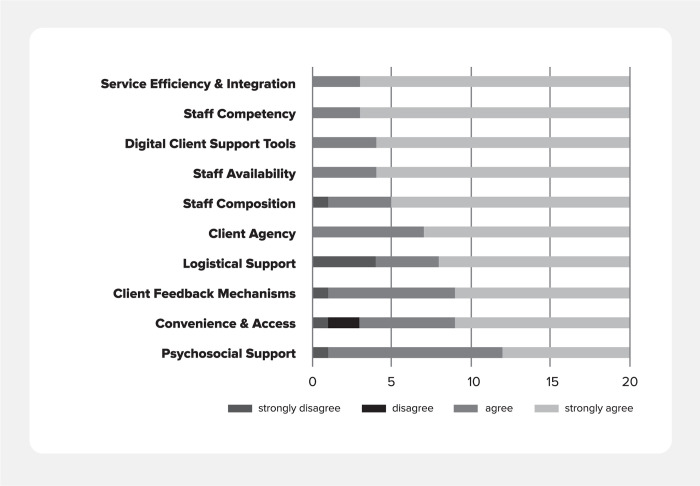
Clients’ degree of agreement on importance of subdomain within PCC framework.

The majority of clients agreed or strongly agreed that all PCC framework domains and subdomains were important to them. On average, clients felt staff competency (3.9) service efficiency and integration (3.9) and digital client support tools (3.8) were most important. Fig 3 summarizes clients’ degree of agreement on importance.

**Fig 4:**
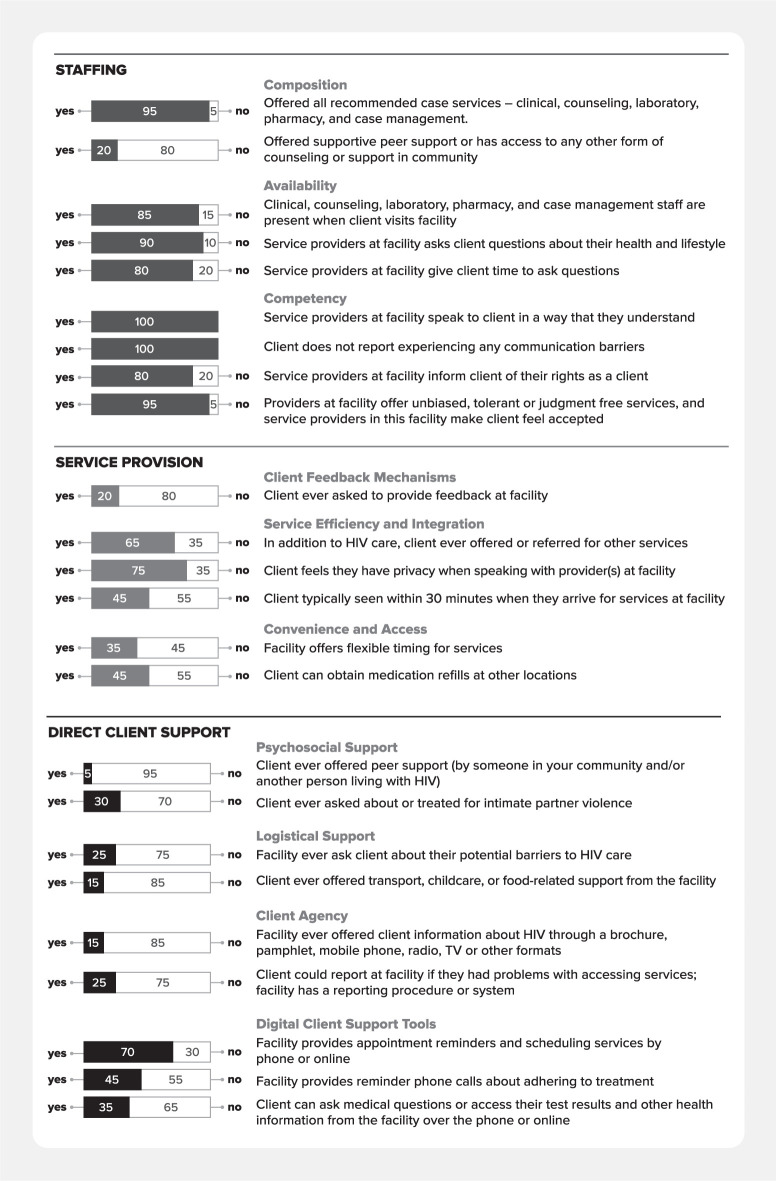
Client perceptions on experiences of care received.

Overall, clients reported that they had service experiences related to care that were in-line with the PCC framework and performance expectations. However, service experience variations emerged within specific domains and subdomains across the five facilities. Overall, clients rated their experiences in care highest within the staffing domain, followed by service provision, and direct client support, with only one service experience area scoring >50% within this domain. Fig 4 summarizes clients’ reported PCC service experiences.

### Key informant interview findings

Coding of KII transcripts uncovered several key themes related to client experiences of PCC (Table 3). It is important to note the interrelated nature of privacy, stigma and discrimination, and infrastructure themes, with clients expressing, for example, that infrastructure contributes to lack of privacy and that it is used as an expression of stigma and discrimination when infrastructure access, such as access to washrooms, is prohibited among clients.

**Table 3:** Key themes related to clients’ experiences with PCC.

Theme	Description	*Corresponding Vignettes*
**Privacy**	Clients expressed concerns when patient education and ART was provided in group settings and when consultation rooms were not private.	‘*Even sometimes the answers you give will be different because you lose focus paying attention to others who may be watching or listening to you’*.*—KII*, *Facility 2*
		‘*Ummm to me I am not shy to come for my medicine but I don’t like how things are structured here because others are shy and I feel like they shouldn’t be giving the medicine in the presence of others*.*—KII*, *Facility 2*
**Stigma and discrimination**	Clients generally reported being treated respectfully with a few exceptions including concerns that ART providers will report their status to others in the community or that staff will unintentionally share their status.	‘*Everyone deserves love*, *everyone needs someone in their life who would make them feel valued as human beings and be treated in that regard*. *Especially for those of us in such conditions*, *we need all the extra love and support we can get from society’*.*—KII*, *Facility 5*
		‘*The last time I went to the lab*, *the technician took my blood sample and said there is another “case” here*, *when he said that all of them stared at me*. *The way he said it made them start staring at me and I know the way he said everyone there at the moment would know the illness I came there with’*.*—KII*, *Facility 2*
**Infrastructure**	Infrastructure was noted by some clients as an issue that contributed to their physical discomfort.	‘*… my only issue is with the facility itself*. *Like the chairs*, *even though we are sick people*, *we should not come here with sickness and leave with another sickness*, *like the rust on the scales could cause harm*. *The conditions in this facility should be looked at in my opinion*. *Even though we are not from here*, *we need to get a comfortable area with better fans*, *water to wash our hands*, *etc*. *We may not get these things at home but when we come here*, *we expect to get these things’*.*—KII*, *Facility 3*
	Infrastructure was closely related to experiences of stigma and discrimination in one facility that did not allow clients to use the washrooms.	‘*…when it’s World AIDS Day*, *they go on radio and do campaigns against stigma but they are practicing it here*. *Why is it that you’d have FOUR washrooms and yet you can’t allow elderly clients to use it’*.*—KII*, *Facility 5*
		‘*I say stigma because they don’t allow us to use their washrooms*. *So they lock it but the nurses and others are allowed to use them…You have to go outside and urinate and people passing will see you*. *Even all the clients*. *When you come here and you have a stomach upset*, *you’ll not be allowed*. *That’s what pains me the most*. *We’re all human beings’*.*—KII*, *Facility 5*
**Logistical Support**	Costs associated with transport and medicine (other than ART) are common and a source of stress.	‘*Okay the medication they give us is very good but the blood tonic*, *the multivitamin*, *we buy it ourselves*. *The prices have increased and there is hardship*. *Septrin is selling for 4 cedis in some areas and I have to buy for my child and me*. *So sometimes when you don’t have money*, *it’s difficult’*.*—KII*, *Facility 5*
	Clients overwhelmingly reported that they are not asked about logistical barriers that may interfere with self-care including taking medicine or accessing services.	‘*Sometimes when you don’t come*, *you’ll be asked why you missed your appointment*, *and you can say maybe you traveled*. *They can’t help you if you tell them you didn’t come because you didn’t have money for transportation*. *They won’t give you the lorry fare’*.*—KII*, *Facility 2*
	Some clients reported receiving financial and food support in the past, including from community organizations, but none reported currently receiving support.	‘*I believe the days when mothers were assisted with bags of rice and other cooking items should be brought back*. *This is because we are in difficult times and most households are unable to afford such items*. *Not only would this ensure that the patients or mothers are well fed as they take their drugs*, *it also serves as an incentive for mothers to come to the facility for their drugs’*.*—KII*, *Facility 5*
**Access to information**	Most clients reported that they could ask their provider questions about their health or call between appointments.	‘*They also give their numbers to the clients to call when they are facing any difficulties*. *We call and they also call’*.*—KII*, *Facility 4*
	Access to VL results was a common concern. Long wait times for VL results increased feelings of anxiety.	‘*My only concern is when I come and do the test for my viral load and I ask for my results*, *they say it’s not available and then I’m asked to take another one*. *It makes me so worried’*.*—KII*, *Facility 4*
		‘*That is my challenge*. *Anytime I ask about it*, *I am told mine has not come yet…They cannot tell if it is lost or not*. *I am very happy you asked about that because I often ask questions about my lab tests and I do not get positive feedback’*.*—KII*, *Facility 3*
**Wait times**	Most clients reported that they are given appointments for a specific day and clients arriving on that day are attended to in the order they arrive.	‘*So maybe if 20 people have the same appointment day as you*, *you wait according to how early you arrive*. *It is on a first come*, *first serve basis’*.*—KII*, *Facility 2*
	12 clients reported that wait times range between 15 minutes to one hour, which they found to be acceptable.	‘*By 8am they are here and within 30mins everything is sorted’*.*—KII*, *Facility 4*
	8 clients reported wait times between 1–6 hours in the morning which led some of them to wait until the afternoon to visit when there are less clients waiting.	‘*When you get here at 8*, *you’re done by 2 O’clock in the afternoon’*.*—KII*, *Facility 5*
		‘*There are some patients who would have to leave and go to their workplaces so it’s necessary that treatment is done in a timely manner for us to leave on time to attend to our daily activities*. *Due to delays caused by long queues*, *some patients choose to come in the afternoon when most nurses have closed from work*. *They miss out on receiving treatment or their drugs’*.*—KII*, *Facility 5*
	Some clients reported that they were seen quickly by the clinician, but that subsequent wait times at the pharmacy were too long.	‘*What I can say is that*, *before patients arrive at the facility the drugs should be prepared and ready for collection*. *If patients are assured of not spending long hours here when coming for treatment or drugs*, *they’ll always show up to the facility*. *The long queues and delays are disincentives for patients coming to the facility’*.*—KII*, *Facility 5*
**Differentiated ART dispensation**	Most clients preferred to pick up their medications at the health facility pharmacy. Some clients expressed that clients who are sick or having adherence challenges should receive home delivery.	‘*Oh*, *with me*, *what I will say is some of my friends when it’s time for them to go for their drugs they don’t*, *others too are not consistent in taking their medication*. *So*, *I will be glad if sometimes they will try and visit us in our homes just to get to know those who are not taking their medication’*.*—KII*, *Facility 3*
		‘*The other time I told her sometimes I’m working and wish my medication could be delivered to me’*.*—KII*, *Facility 1*
	Three clients reported being able to pick up their medication at other pharmacies that are convenient.	‘*When I’m not able to make it here for my appointment*, *I can go to any other facility with my appointment card and I’ll get it*. *Because it is system based*, *when you come with your appointment card and you say you’ve gone somewhere else*, *they’ll verify from their system’*.*—KII*, *Facility 5*
	Three clients reported flexibility in how they receive their medication with either home delivery by a nurse or NGO staff, or community collection.	‘*Okay so when it’s time for me to come for my medication and I’m unable to*, *I give my card to [NGO peer support] and she picks them up for me*. *So when I’m ready*, *I go to her house to pick it up’*.*—KII*, *Facility 2*
**Client feedback**	Six clients reported that they had either been asked about their experiences by a provider, been offered a phone number, WhatsApp, or been reached out to by an NGO for feedback.	*‘But because I have it in mind*, *I know where to lodge complaints and report when my rights are being infringed upon’*.*—KII*, *Facility 5*
	14 clients reported that they were not aware of any client feedback mechanisms	*‘It (client feedback) is important because “the one paving the way doesn’t know it is curved unless they are told by someone behind*.*” So when they’re not doing the right thing you tell them that they’ll adjust themselves so it’s good’*.*—KII*, *Facility 4*
**Client suggestions for improving PCC**	Service friendliness	‘*People must learn how to interact with others politely instead of being harsh*. *For instance “mummy*, *please remember to refill your medication when it is almost done”*, *but some nurses will say this in a very disrespectful manner*, *which is not encouraging at all’*.*—KII*, *Facility 3*
	Drug formulations	‘*I like the drugs I’m given but if it can be changed to an injection*, *it will help because sometimes I forget’*.*—KII*, *Facility 1*
	Peer counseling	‘*During my time*, *[NGO peer support] would call me everyday to ask how I’m doing and encourage me*. *So if someone is in that situation*, *especially in the beginning*, *they must receive more counseling and encouragement*. *It will help a lot’*.*—KII*, *Facility 3*
	Virtual adherence support	‘*I think calling the patient to find out her state of health and to ask whether she has taken her medication*, *will serve as motivation for the patient to take her drugs since most of the patients do not take the drugs as they should*. *It also demonstrates love and care on the part of the health workers*, *and this is a way for patients and caregivers to draw closer to one another*. *This will influence patients positively to take their drugs’*.*—KII*, *Facility 5*

## Discussion

The primary purposes of this study were to test the scoring consistency and reliability of the PCC-AT by determining if ART providers and client perspectives on PCC services would be similar. Although the sample size was small and none of the correlations were significant the data shows alignment between the client and providers’ perspective that can be further explored in future studies. It is important to note that in four out of the five facilities, clients tended to score PCC performance higher than ART providers demonstrating that ART providers tended to rate their performance more critically than their clients. This, in part, may be attributed to the fact that ART providers were rating their performance based upon specific performance expectations while client’s ratings were more subjective in nature. Given the objective of the PCC-AT is for ART providers to assess their own PCC performance and to develop numerical thresholds that would prompt improvement plans, a tendency towards more critical scoring would be the preferred trend. These findings show the PCC-AT can provide consistent and reliable scoring that prompts PCC improvement activities at an appropriate threshold level.

A secondary purpose was to determine if the domains and subdomains of the PCC-framework were in-line with clients’ perspectives and priorities for PCC. This exercise intended to ensure that the performance expectations within the PCC-AT were indeed measuring aspects of person-centered care that are meaningful to diverse clients. Almost overwhelmingly, clients agreed that domains and subdomains present within the PCC framework are important. Of note, clients rated the subdomains of service efficiency and integration, and digital client support tools as the top first and third most important aspects of care, relatively. However, they also reported notable gaps in care aspects that they actually received within these subdomains, highlighting priorities for improvement activities.

Notable within our KII findings was that rarely were clients asked to provide feedback to their ART providers regarding service-related challenges and concerns, and those clients that were aware of feedback mechanisms scarcely utilized them. Some clients expressed hesitancy to provide feedback either due to feeling shy, or not wanting to be perceived as bothersome. Most clients stated that client feedback mechanisms are either important or very important. Given the very foundation of person-centered care relies on inputs from clients who provide meaningful information that helps to shape the services, increasing client confidence to use the reporting mechanisms is an important measure to strengthen overall PCC [23].

KII findings on client suggestions for PCC service improvements are in-line with the larger research base in terms of enhancing the friendliness of services for diverse populations [24, 25], and enhancing adherence support through peer counselors [26], digital outreach [27, 28] and more convenient drug formulations [29, 30]. Unexpectedly, many clients reported that they do not need logistical support such as transportation vouchers, childcare support and other logistics that enable service access. Many also stated that they prefer to collect ART at the health facility. This is surprising given that previous research shows an association between differentiated ART delivery models and cost savings for clients who can access medication at alternative locations [31, 32]. In many of the KIIs though, clients stated that although they do not require logistical support and/or flexibility in ART access, these mechanisms should be in place for clients who are struggling financially, have adherence challenges, or have other concerns such as being sick or cannot leave work.

This study has several limitations. While the study team noted repetition of themes among participants indicating progress towards saturation, the individuals were not randomly selected, and a larger and non-convenience sample may have elicited different information. Selection of participants for KII was not random and client participants were largely composed of women which increases the risk for bias among our sample of clients. Also present among ART providers and client FGDs and KIIs is a risk for response biases related to perceived benefit or negative consequence. Facility infrastructure presented challenges wherein four out of the five facilities, interviews were conducted in consultation rooms which were interrupted at times, limiting privacy and attention.

## Conclusion

Study findings indicate that the PCC framework is in-line with PCC priorities for clients assessing HIV treatment services, and two out of the three domains demonstrated consistency between ART providers and client PCC scores. Future research should systematize the PCC-AT process to integrate client perspectives that inform PCC-AT adaptation prior to implementation. A follow-up study in Zambia with an additional project team will integrate learnings from this study to determine if higher PCC-AT scores result in improved clinical outcomes among clients.

## Supporting information

S1 File Description of PCC-AT Domain and Sub-domains. (DOCX)

S2 File Health facility characteristics. (DOCX)

S3 File KII Guide. (DOCX)

S1 Data Dataset. (XLSX)
