# Supplementary material for: Do HIV provider and client perspectives align on person-centered care? Lessons learned from implementation of the Person-Centered Care Assessment Tool (PCC-AT) in HIV treatment settings in Ghana
Source: PLOS Glob Public Health. 2024 Sep 6;4(9):e0003457. doi: 10.1371/journal.pgph.0003457 (PMC11379259; doi:10.1371/journal.pgph.0003457)
Supplement: S1 File — (DOCX) [file pgph.0003457.s001.docx]

**Supplement 1. Description of PCC-AT Domain and Sub-domains**


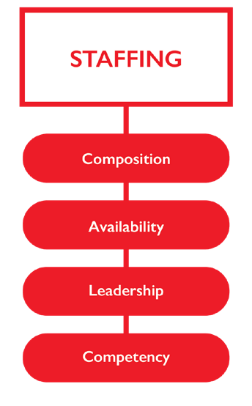
**Staffing** is key to ensure that we are collaborating with clients to gather medical, personal, and social histories; make shared decisions; and document clients’ preferences to allow for transparency and to facilitate continuity of care. The subdomains examine the mix of qualified staff positions offering the required clinical, counseling, laboratory, pharmacy, and case management services required for optimal client care for target populations. They also examine if staff allocation meets national standards (per catchment area population) to maximize service delivery and reduce wait times, and if facility staff understand the unique needs of HIV-affected individuals and populations while offering culturally-appropriate, individualized tailored care based upon each person’s needs.


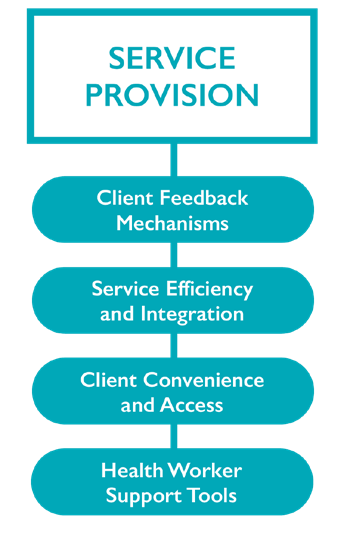
**Service provision** focuses on attributes of integrated and coordinated care that include respect for a person’s preferences. The subdomains examine if multiple mechanisms exist for individuals, families, and community organizations to provide input and feedback in a routine and systematic manner. They also examine if facilities arrange and sequence services for optimal flow – in terms of speed, efficiency and service integration—and if they provide differential and tailored care based on the person’s needs, offering a range of options that meet frequency, intensity, location, and timing of service delivery to address access issues. The use of digital tools is additionally checked to ensure the exchange of information between health service providers, support clinical decision making for providers, and offer alternative person-provider communication platforms.


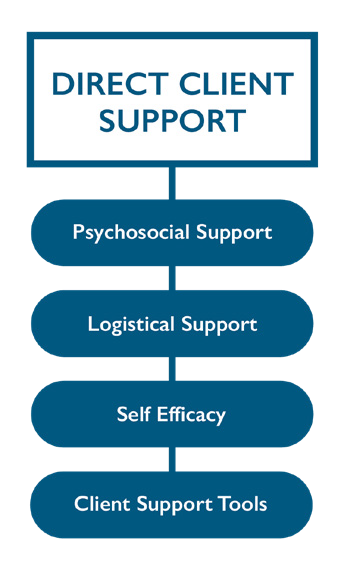
**Direct client support** focuses on building agency through client information and education, emotional and logistical support, and family and friend involvement. The subdomains include community-based psychosocial support services addressing the mental, emotional, and social needs of people and their families or other trusted social support networks. The domains also examine if health facilities consistently identify vulnerable individuals and provide logistical support for common barriers to care such as transportation, food supplementation, and child care. Further, if facilities build self-management and agency through offering tools for clients to both understand their health condition and exercise their rights to live a better life with HIV are included. Of course, tools that safeguard privacy and streamline access to information about health conditions, medical services, and appointments are also considered.
