# Supplementary material for: Do HIV provider and client perspectives align on person-centered care? Lessons learned from implementation of the Person-Centered Care Assessment Tool (PCC-AT) in HIV treatment settings in Ghana
Source: PLOS Glob Public Health. 2024 Sep 6;4(9):e0003457. doi: 10.1371/journal.pgph.0003457 (PMC11379259; doi:10.1371/journal.pgph.0003457)
Supplement: S2 File — (DOCX) [file pgph.0003457.s002.docx]

**Supplement 2: Health facility characteristics**

| **Facility** | **Caseload (as of May 2023)** | **Type** | **Ownership** | **Geography** |
| --- | --- | --- | --- | --- |
| **1** | **54** | **Health Center** | **Private-public** | **Urban** |
| **2** | **702** | **Hospital** | **Government/public** | **Urban** |
| **3** | **315** | **Health Center** | **Government/public** | **Rural/ peri-urban** |
| **4** | **275** | **Hospital** | **Government/public** | **Urban** |
| **5** | **2,256** | **Referral hospital** | **Government/public** | **Urban** |
