# Supplementary material for: Do HIV provider and client perspectives align on person-centered care? Lessons learned from implementation of the Person-Centered Care Assessment Tool (PCC-AT) in HIV treatment settings in Ghana
Source: PLOS Glob Public Health. 2024 Sep 6;4(9):e0003457. doi: 10.1371/journal.pgph.0003457 (PMC11379259; doi:10.1371/journal.pgph.0003457)
Supplement: S3 File — (DOCX) [file pgph.0003457.s003.docx]

**Supplement 3: KII Guide**

***Introduction:***

Hello, my name is ___ and I am a researcher for JSI conducting a study on person-centered care tool validation. We would like to take a moment of your time to get an overall sense of the care you receive here as well as your reactions to the results of an assessment we recently completed at this facility.

**Instructions:** review participant sheet and consent form

**Eligibility question:**

Are you a current client of this facility receiving HIV care/treatment?

**What we did:**

We sat with key staff at this facility and reviewed a set of questions that are intended to measure person centered care at this facility. Person-centered care (PCC) is a component of ‘quality of care’ that moves beyond clinical quality of care to include concepts such as support, respect, and autonomy.

We would now like to ask you a few questions about your experience accessing care at this facility.

***Questions for PLHIV: (clients, KII)***

**Experience of care received:**

Domain: Staffing

- *Subdomain: Composition*
  - At this facility, have you been offered all recommended case services – clinical, counseling, laboratory, pharmacy, and case management? Have you ever been offered supportive peer support? *(probe: expert client, navigators from the community)*
- *Subdomain: Availability*
  - Are clinical, counseling, laboratory, pharmacy, and case management staff present when you visit the facility?
  - Do service providers at this facility ask you questions about your health and lifestyle?
  - Do service providers at this facility give you time to ask questions?
- *Subdomain: Competency*
  - Do service providers at this facility speak to you in a way that you understand? (*note: cultural and linguistic proficiency*)
  - Do service providers at this facility inform you of your rights as a client here (for example, that you have equal access to services)?
  - Do you feel providers at this facility offer unbiased, tolerant or judgment free services?

Domain: Service Provision

- *Subdomain: Client Feedback Mechanisms*
  - Have you ever been asked to provide feedback at this facility? If yes, what kind? (*probe: suggestion box, call-in number, post-care survey, or participation in a committee or meeting to give feedback on services to facility staff*)
  - [If yes to the above], have you noticed any actions taken based on your feedback?
- *Subdomain: Service Efficiency and Integration*
  - In addition to your HIV care, have you ever been offered or referred for other services? (*probe: TB, FP, MNCH, NCD, mental health and substance use services*)
  - Do you feel that you have privacy when speaking with provider(s) at this facility, or do you worry that you can be overheard or seen by others?
  - When you arrive for services at this facility, are you typically seen within 30 minutes, or do you have to wait a long time?
- *Subdomain: Convenience and Access*
  - Does the facility offer flexible timing for services? (*probe: during weekends or evenings*)
  - Can you obtain medication refills at other locations? (*probe: mobile/outreach/community ART, private pharmacy, home delivery, other special locations*)

Domain: Direct Client Support

- *Subdomain: Psychosocial Support*
  - Have you ever been offered peer support (by someone in your community and/or another person living with HIV)?
  - Have you ever been asked about or treated for intimate partner violence?
- *Subdomain: Logistical Support*
  - Has the facility ever asked you about your potential barriers to HIV care? (*probe: lack of transport, childcare barriers, food insecurity, among others*)
  - Have you ever been offered transport, childcare, or food-related support from the facility?
- *Subdomain: Client Agency*
  - Has the facility ever offered you information about HIV through a brochure, pamphlet, mobile phone, radio, TV or other formats?
    - If yes, were they easy to understand? (*probe: language, images/graphics, simple terminology*)
  - If you wanted to report problems with accessing services, could you report at the facility? Do they have a reporting procedure or system?
- *Subdomain: Digital Client Support Tools*
  - Does the facility provide appointment reminders and scheduling services by phone or online?
  - Does the facility provide reminder phone calls about adhering to treatment? For example, will they call you if your medication is not collected?
  - Can you ask medical questions or access your test results and other health information from the facility over the phone or online?

**Impressions of PCC at facility:**

- How long have you been on treatment?
- *Person centered care is described as…* Person-centered care (PCC) is a component of ‘quality of care’ that moves beyond clinical quality of care to include concepts such as support, respect, and autonomy… *Do you feel you receive PCC services at this facility?*
- What do you think of the care you receive at this facility?
- What aspects do you like the most?
- What areas do you feel need improvement?
